# Consequence of temperature changes on cercarial shedding from *Galba truncatula* infected with *Fasciola hepatica* or *Paramphistomum daubneyi*

**DOI:** 10.1051/parasite/2013009

**Published:** 2013-03-19

**Authors:** Daniel Rondelaud, Amal Titi, Philippe Vignoles, Abdeslam Mekroud, Gilles Dreyfuss

**Affiliations:** 1 INSERM U 1094, Faculties of Medicine and Pharmacy 87025 Limoges France; 2 PADESCA Laboratory, Veterinary Science Institute 25100 El-Khroub Constantine Algeria

**Keywords:** Cercaria, Cercarial shedding, *Fasciola hepatica*, *Galba truncatula*, *Paramphistomum daubneyi*, Temperature change

## Abstract

Experimental infections of *Galba truncatula* (two populations) with *Fasciola hepatica* or *Paramphistomum daubneyi* were carried out to study the effect of water temperature changes (3 h at a mean of 12 °C every week) on cercarial shedding during the patent period. The results were compared with those of control snails infected according to the same protocol and always maintained at 20 °C. Compared to controls, a significant increase in the number of cercariae-shedding snails, a significantly longer patent period and significantly greater cercarial production were noted in temperature-challenged snails, regardless of the type of digenean infection. In contrast, the number of incompletely formed metacercariae was significantly higher in temperature-challenged snails than in controls. Incompletely formed metacercariae of *F. hepatica* consisted of cysts whose colour remained whitish after shedding (25.4% for temperature-challenged snails) or whose dome was flattened after encystment (74.6%). Those of *P. daubneyi* were totally dark brown or blackish after formation. These incomplete metacercariae might originate from young differentiating cercariae within the snail body (*F. hepatica*) or from cercariae which died just after encystment (*P. daubneyi*). The use of regular temperature changes for snails infected with *F. hepatica* or *P. daubneyi* must be monitored carefully during collection of metacercariae to select completely formed cysts for infecting definitive hosts.

## Abstract (fr)

Des infestations expérimentales de *Galba truncatula* (2 populations) avec *Fasciola hepatica* ou *Paramphistomum daubneyi* ont été réalisées pour étudier l’effet des changements de température (3 heures à 12 °C en moyenne chaque semaine) sur les émissions cercariennes durant la période patente. Les résultats ont été comparés avec ceux provenant de témoins infestés selon le même protocole et maintenus toujours à 20 °C. Par rapport aux témoins, un accroissement numérique significatif des limnées émettant des cercaires, une période patente significativement plus longue et une production cercarienne significativement plus élevée ont été notés chez les limnées soumises aux changements de température quelle que soit l’espèce du Digène. Par contre, les métacercaires incomplètement formées sont significativement plus nombreuses chez les limnées soumise aux changements de température que chez les témoins. Dans le cas de *F. hepatica*, ces métacercaires incomplètement formées concernent des kystes dont la couleur reste toujours blanchâtre après l’émission (25,4 % chez les limnées soumises aux changements de température) ou dont le dôme s’affaisse après l’enkystement (74,6 %). Celles de *P. daubneyi* sont totalement marron foncé ou noirâtres après la formation des kystes. Ces métacercaires incomplètes pourraient provenir de jeunes cercaires en cours de différenciation dans le corps du mollusque (*F. hepatica*) ou de cercaires qui meurent juste après leur enkystement (*P. daubneyi*). L’utilisation de changements réguliers de température chez les mollusques infestés par *F. hepatica* ou *P. daubneyi* nécessite d’être très attentif lors de la récolte des métacercaires afin de ne sélectionner que des kystes complètement formés pour l’infestation des hôtes définitifs.

## Introduction

Different factors (reviewed by Smyth & Halton [15]) may have an influence on the emergence of cercariae from their snail hosts. Among these factors, temperature and light are often cited. However, the influence of these factors is also trematode-specific. In the model *Galba truncatula-Fasciola hepatica*, the release of cercariae was optimum at a constant temperature of 20 °C (for snail populations highly susceptible to the parasite) and decreased when the temperature was lowered [4, 8, 9]. In contrast, in the model *G. truncatula-Paramphistomum daubneyi*, only few cercariae emerged from *G. truncatula* at 20 °C and occurred only if infected snails were subjected to temperatures fluctuating daily from 6–8 °C to 20 °C [1, 2]. In both cases, the number of snails containing cercariae but dying without emission remained low at 20 °C (*F. hepatica*) or during fluctuations from 6–8 °C to 20 °C (*P. daubneyi*), and was significantly higher for opposite temperatures (20 °C, for example, for *P. daubneyi*).

The use of regular temperature changes to induce cercarial release from infected snails has already been used in the case of Lymnaeidae. However, the conditions used to cause these temperature changes are variable. Infected lymnaeids can be placed on ice [5], in the refrigerator [11] or in 6–8 °C water during each daily change [1, 2]. The use of such low temperatures, even during a short time, may cause mortality of infected snails which are generally less resistant to environmental changes than uninfected ones [13]. Moreover, the repetitive use of this method over time did not always give the same results for shedding because *F. hepatica* cercariae, for example, did not mature at the same time [3] and there was a 6–8 day periodicity in cercarial shedding for some snails infected with this digenean [18]. In view of these data, a change in water temperature, compatible with natural fluctuations of this parameter existing in snail habitats in the spring, would be interesting to study its effects and its consequences on cercarial shedding from *G. truncatula*. To verify this possibility, snails experimentally infected with *F. hepatica* or *P. daubneyi* were subjected during the patent period to a constant temperature of 20 °C, or a temperature change every week (snail dishes were placed at a mean temperature of 12 °C during 3 h before being replaced at 20 °C).

## Materials and methods

### Snails and parasites

Both *G. truncatula* populations (A) and (B) were chosen on the fact that high numbers of snails died without emission (in spite of numerous free cercariae within snail bodies) when experimentally infected with local miracidia of *F. hepatica* at 20 °C [17]. Their habitats were road ditches on the respective communes of Châteauponsac (46°8′2″ N, 1°17′30″ E) and Rancon (46°6′54″ N, 1°10°34″ E), department of Haute Vienne, central France. As these sites were located on acid soil, the maximum height of adult snails ranged from 8 to 9 mm. Three hundred snails, measuring 4 mm in height and belonging to the spring generation, were collected from each site. Thirty adult snails for each site were also dissected under a stereomicroscope to verify the absence of trematode larval forms within their body at the time of their collection. After their collection, snails were kept in the laboratory at 20 °C during 48 h for temperature acclimatization before being exposed to miracidia.

*Fasciola hepatica* eggs were collected from the gall bladders of heavily infected cattle at the slaughterhouse of Limoges, department of Haute Vienne. To obtain *P. daubneyi* eggs, adult worms were collected from the rumen of the same cattle and placed in a saline solution (NaCl, 0.9%; glucose, 0.45%) for 4 h at 37 °C. All eggs were washed several times with spring water and were incubated at 20 °C for 20 days in the dark in order to obtain miracidia [12].

### Experimental protocol

Four hundred snails (Table 1) were individually exposed to *F. hepatica* (two miracidia per snail for 4 h at 20 °C in 3.5 mL of spring water). The same protocol was used for the other 200 snails exposed to *P. daubneyi*. They were then individually raised in 35-mm Petri dishes (volume of spring water, 3.5 mL) according to the method by Rondelaud et al. [14]. Snails were fed with a piece of dried lettuce leaf and another of dead grass (*Molinia caerulea*), while oxygenation of the water layer was ensured by a piece of live spring moss (*Fontinalis sp.*). The dissolved calcium in spring water was 35 mg/L. Petri dishes were placed in an air-conditioned room at a constant temperature of 20 °C (±1 °C) and a diurnal photophase of 10 h involving a light intensity of 3,000–4,000 lux over dish tips.

**Table 1.** Experimental protocol used to study the effect of thermal shock on production of *F. hepatica* or *P. daubneyi* cercariae. TC, temperature-challenged.

Snail population and parasite	Number of snails at exposure	Subgroups at day 30 p.e.		
		Water temperature	Number of snails per subgroup	Physiological state of snails
(A)				
*F. hepatica*	200	20 °C constantly	81	Controls
		Thermal shock	81	TC snails
*P. daubneyi*	100	20 °C constantly	44	Controls
		Thermal shock	45	TC snails
(B)				
*F. hepatica*	200	20 °C constantly	77	Controls
		Thermal shock	78	TC snails
*P. daubneyi*	100	20 °C constantly	39	Controls
		Thermal shock	40	TC snails

At day 30 post-exposure (p.e.), the surviving snails from each group were divided into two subgroups, as indicated in Table 1. Spring water and food were changed between 1 p.m. and 5 p.m. if necessary. When the first cercarial shedding occurred, the Petri dishes containing snails of four subgroups (temperature-challenged snails) were placed every week at a mean temperature of 12 °C (minimum-maximum, 10–14 °C) for 3 h (8–11 a.m.) under the same lighting conditions (3,000–4,000 lux) and were then replaced at 20 °C during the rest of the week. The mean temperature of 12 °C was chosen because most cercariae in the case of *F. hepatica* did not emerge from snails below 10 °C [9], while the reason for selecting a weekly interval for temperature changes was the 6–8 day periodicity that Vignoles et al. [18] have reported in cercarial shedding for some snails infected with *F. hepatica*. To induce thermal shock, the morning was chosen because water temperature in Petri dishes fell from 20 °C to 10–11 °C in 20–25 min (when these recipients were placed outdoors at this temperature at 8 a.m.) and progressively increased at 14 °C with increasing air temperature (generally reached at 11 a.m.). Contrary to temperature-challenged snails, *G. truncatula* from the other four subgroups (controls, Table 1) were always kept at a constant temperature of 20 °C. The above protocol was followed during 11 weeks in the case of *F. hepatica* and 9 weeks for *P. daubneyi* according to the length of the patent period. If metacercariae (mcc) were present in a Petri dish, the snail was placed in a second dish with its food, while mcc of the first dish were counted two days later and classified according to their type (floating mcc, fixed mcc, each having a complete cyst, and fixed and incomplete mcc) before their removal. In the case of *F. hepatica*, two categories of fixed and incomplete mcc were considered: (i) whitish cysts whose colour had not become flaxen 2 days after shedding, and (ii) whitish cysts whose dome became flattened after its formation. In contrast, fixed and incomplete mcc of *P. daubneyi* were completely dark brown or blackish just after cercarial encystment, while fixed and complete mcc were refringent with a peripheral brown-blackish ring [13].

A routine post-mortem dissection of snail cadavers was performed to separate uninfected snails and those containing only rediae from snails which contained free cercariae and died without shedding (NCS snails).

### Parameters studied

The first two were the frequency of cercariae-shedding snails (CS snails) and that of NCS snails. In each group, the frequencies were calculated in relation to the number of snails surviving at day 30 p.e. Another parameter was prevalence of infection determined in each group by adding the number of CS snails and that of NCS snails. The differences between frequencies of CS snails, those of NCS snails and prevalences of snail infection were compared using a χ^2^ test. The last parameters were the growth of CS snails during experiment (between snail exposure to miracidia and death), length of the prepatent period, that of the patent period and the number of mcc according to their type (floating, complete and fixed, or incomplete and fixed mcc). One- or two-way analysis of variance was used to establish statistical levels of significance for these last parameters. All statistical analyses were done using the Statview 5.0 software.

The distribution of complete or incomplete and fixed mcc throughout the length of the patent period (expressed in weeks) was also determined. Floating mcc were not considered for this study because most of them were present only during the first 2 weeks.

## Results

### Snails infected with *F. hepatica*

Compared to controls which were always maintained at a constant temperature (Table 2), the frequency of CS snails was significantly increased [population (A): χ^2^ = 29.84, *p* < 0.001; population (B): χ^2^ = 17.68, *p* < 0.001)] in temperature-challenged groups. However, when each population was considered separately, prevalence values were close to each other and no significant difference was noted. The differences between shell growths of CS snails during the experiment as well as between prepatent periods were insignificant, regardless of snail population and group. In contrast, the patent periods noted for temperature-challenged snails were significantly longer (*F* = 4.12, *p* < 0.01) than for controls, while snail population did not have an effect on this parameter. The number of floating mcc varied significantly (*F* = 3.55, *p* < 0.05) with the snail population but was not clearly affected by temperature change. In the other two mcc categories, the values noted for temperature-challenged snails were significantly greater (fixed and complete mcc: *F* = 6.74, *p* < 0.01; fixed and incomplete mcc: *F* = 11.52, *p* < 0.001) than those recorded for controls, while the effect of snail population was insignificant. Total *F. hepatica* mcc production per CS snail, including floating mcc, ranged from 143.3 in controls (B) to 361.6 in temperature-challenged snails (A). In contrast, if this production was correlated with the number of *G. truncatula* at day 30 p.e., the mean values were clearly different between controls (26.0–58.5 mcc) and temperature-challenged snails (118.1–218.7).

**Table 2.** Main characteristics of *F. hepatica* infection in snails maintained to a constant temperature of 20 °C (controls), or subjected every week to a 3-h stay at a mean temperature of 12 °C (TC, temperature-challenged; CS, cercariae-shedding; *SD*, standard deviation).

Parameters	Population (A)		Population (B)	
	Controls	TC snails	Controls	TC snails
Number of snails at day 30 p.e.	81	81	77	78
Number (frequency in %):				
CS snails	23 (28.3)	49 (60.4)*	14 (18.1)	37 (47.4)*
NCS snails	35 (43.2)	6 (7.4)	24 (31.1)	9 (11.5)
Prevalence of infection (%)	71.6	67.9	49.3	58.9
Mean growth (*SD*) of CS snails during experiment (mm)	3.2 (0.8)	3.1 (1.0)	2.3 (0.7)	2.3 (0.9)
Mean length (*SD*) in days:				
Prepatent period	49.5 (7.7)	48.3 (3.0)	50.2 (8.3)	48.2 (3.1)
Patent period	30.7 (9.2)	46.2 (10.3)*	24.5 (11.5)	38.7 (8.9)*
Mean number (*SD*) of mcc shed by CS snails				
Floating mcc	11.5 (5.3)	13.3 (4.8)	7.2 (3.1)	4.5 (1.7)
Fixed and complete mcc	217.1 (69.3)	304.0 (80.5)*	134.6 (50.4)	214.0 (71.0)*
Fixed and incomplete mcc	3.2 (2.4)	44.3 (27.2)*	1.5 (0.7)	30.6 (11.4)*
Total mcc production:				
Per CS snail	231.8	361.6	143.3	249.1
Per snail at day 30 p.e.	58.5	218.7	26.0	118.1

* Significant differences between temperature-challenged snails and controls.

If the values recorded for incomplete and fixed mcc in both populations of snails were pooled, cysts that always remained whitish represented 100% of mcc in controls and 25.4% in temperature-challenged snails. In contrast, whitish mcc with a flattened cyst and often visible cracks in their outer wall (74.6%) were only observed in temperature-challenged snails (data not shown).

**Figure 1.**
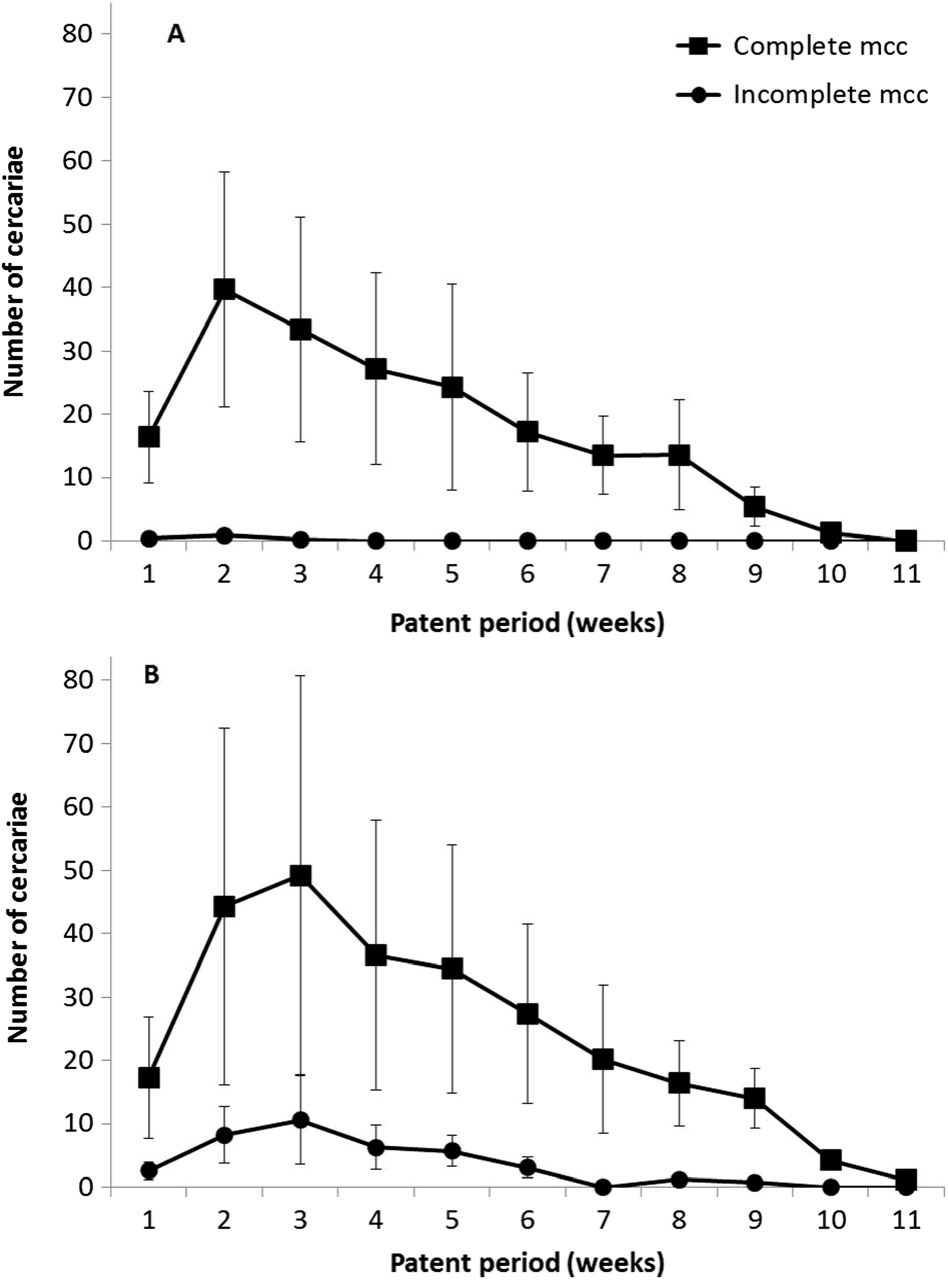
Distribution of *F. hepatica* metacercariae (mcc) for controls (A) and temperature-challenged snails (B) correlated to length of patent period (expressed in weeks). The values noted for controls from both snail populations were pooled. A similar process was also used for values coming from temperature-challenged snails.

Figure 1 shows the distribution of fixed mcc in relation to patent period length expressed in weeks. The number of complete mcc peaked during week 2 for controls and week 3 for temperature-challenged snails, and decreased thereafter in both subgroups until week 11. In contrast, the distribution of incomplete mcc varied with the snail group because they were seen during the first three weeks of the patent period in controls and the first nine weeks in temperature-challenged snails (apart from week 7 for which no incomplete and fixed mcc were noted).

### Snails infected with *P. daubneyi*

Table 3 gives the main characteristics of *P. daubneyi* infection in controls and temperature-challenged snails for both populations. In each snail population considered separately, the number of CS *G. truncatula* was significantly higher [population (A): χ^2^ = 42.08, *p* < 0.001; population (B): χ^2^ = 11.91, *p* < 0.001)] in temperature-challenged groups than in controls. In contrast, the difference between prevalence values in each population was not significant. Similar findings were also noted for CS snails shell growth during the experiment and prepatent periods. Compared to controls, the length of the patent period in temperature-challenged groups was significantly higher (*F* = 3.65, *p* < 0.05), whereas snail population did not have a clear effect on this parameter. Significantly greater numbers of fixed mcc (complete cysts: *F* = 4.16, *p* < 0.01; incomplete cysts: *F* = 3.74, *p* < 0.05) were observed in temperature-challenged groups and these results were independent of snail population. In contrast, the differences between floating mcc were insignificant, regardless of snail population and group. If CS snails were considered, the mean total mcc production ranged from 77.1 to 211.9 but was clearly different between controls (14.6–15.8) and temperature-challenged snails (85.9–178.9) if values were correlated to *G. truncatula* numbers at day 30 p.e.

**Table 3.** Main characteristics of *P. daubneyi* infection in snails maintained to a constant temperature of 20 °C (controls), or subjected every week to a 3-h stay at a mean temperature of 12 °C (TC, temperature-challenged; CS, cercariae-shedding; *SD*, standard deviation).

Parameters	Population (A)		Population (B)	
	Controls	TC snails	Controls	TC snails
Number of snails at day 30 p.e.	44	45	39	40
Number (frequency in %):				
CS snails	7 (15.9)	38 (84.4)*	8 (20.5)	21 (52.5)*
NCS snails	31 (70.4)	4 (8.8)	13 (33.3)	3 (7.5)
Prevalence of infection (%)	86.3	93.3	53.8	60.0
Mean growth (*SD*) of CS snails during experiment (mm)	3.3 (1.1)	3.5 (1.0)	2.1 (0.9)	2.3 (0.8)
Mean length (*SD*) in days:				
Prepatent period	69.5 (10.6)	68.7 (11.2)	70.3 (8.0)	68.4 (8.9)
Patent period	27.6 (12.9)	41.5 (7.3)*	24.1 (10.3)	36.7 (6.8)*
Mean number (*SD*):				
Floating mcc	0.7 (0.4)	1.5 (0.9)	1.3 (0.7)	2.4 (1.0)
Fixed and complete mcc	91.4 (47.2)	204.8 (81.0)*	74.6 (38.5)	156.4 (62.3)*
Fixed and incomplete mcc	0	5.6 (1.7)*	1.2 (0.5)	4.8 (1.2)*
Total mcc production:				
Per CS snail	92.1	211.9	77.1	163.6
Per snail at day 30 p.e.	14.6	178.9	15.8	85.9

* Significant differences between temperature-challenged snails and controls.

**Figure 2.**
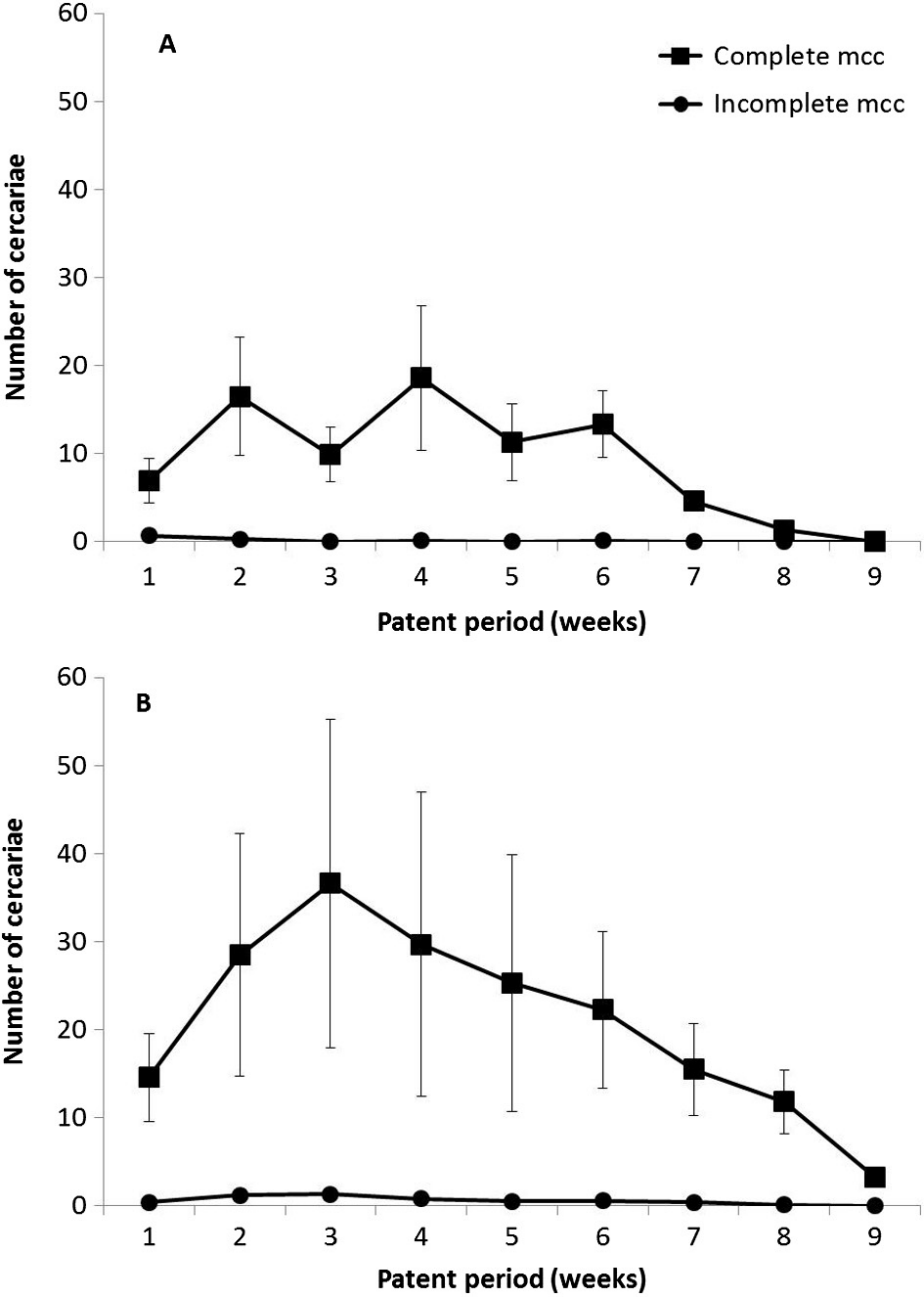
Distribution of *P. daubneyi* metacercariae (mcc) for controls (A) and temperature-challenged snails (B) correlated to length of patent period (expressed in weeks). The values noted for controls from both snail populations were pooled. A similar process was also used for values coming from temperature-challenged snails.

Figure 2 gives the distribution of fixed mcc during the patent period. In complete mcc, peaks were noted during week 4 for controls and week 3 for temperature-challenged snails. In contrast, the distribution of incomplete mcc was similar in both subgroups.

## Discussion

In snail groups subjected every week to a temperature change, a significant increase in numbers of CS snails was noted, regardless of snail population and digenean infection. For *F. hepatica,* temperature change provided an effective stimulus to induce cercarial release in *G. truncatula* populations that were only weakly susceptible to this digenean. Numerous infected snails, which normally kept cercariae within their bodies at a constant temperature of 20 °C, shed these larvae when they were placed at a lower temperature during a few hours. This finding disagreed with reports by Audousset et al. [4], Kendall and McCullough [9] because these authors declared that cercarial release of *F. hepatica* in highly susceptible *G. truncatula* populations was optimum at 20 °C. Two complementary hypotheses may be proposed to explain this difference. The first might be the difference which exists between susceptibilities of snail populations towards the parasite (weakly susceptible in the present study, and highly susceptible in the case of the above authors). The second is to assume that 20 °C would not constitute an ideal temperature for *F. hepatica*-infected snails, even though a quicker development of larval forms was seen than for lower temperatures [12]. An element supporting the latter hypothesis was the number of mcc noted in temperature-challenged snails which was significantly higher than that recorded in controls always raised at 20 °C. The results noted for *P. daubneyi* in the present study agreed with the reports by Abrous et al. [1, 2] and demonstrated that regular fluctuations of temperature are essential for cercarial shedding from numerous infected snails.

The longer patent period in temperature-challenged snails can be easily explained by the protocol used, as the fact of decreasing water temperature, even during a short time, for snails after their first cercarial shedding resulted in a slower differentiation of cercariae over time [12] and, consequently, in lengthened snail life [16]. In contrast, the significantly greater mcc production noted in temperature-challenged snails is more difficult to understand, at least for *F. hepatica*. In our opinion, two perhaps complementary hypotheses might be proposed. The first is to correlate this higher mcc production to the longer patent period seen in temperature-challenged snails, which would allow the release of additional mcc when compared to that noted in controls. The second hypothesis is to interpret this higher mcc production as the consequence of temperature changes because the mean cercarial production of each digenean in temperature-challenged snails (Figures 1 and 2) was clearly higher during the first 6 weeks of the patent period than of control *G. truncatula*. The stimulus caused by temperature change would result in an exit of numerous mature cercariae, followed by some immature cercariae free within the snail but which have not ended their differentiation by accumulating glycogen and fatty acids [7] in their body.

In both digenean infections, incompletely formed mcc were more numerous in temperature-challenged groups than in controls. In the case of *F. hepatica*, mcc which remained always whitish (without flaxen or tawny colour) might correspond to immature cercariae, each having still incompletely differentiated cystogenic cells [6, 10] at the time of shedding. Other incomplete mcc might correspond to younger cercariae in differentiation but these larvae would not support encystment and would die after this process, thus leading to the collapse of their dome-shaped cyst and probably cracking of their outer wall. The formation of these incomplete mcc poses a problem for collecting viable *F. hepatica* mcc coming from temperature-challenged snails because they represented 12.2% (population A) or 14.2% (population B) of mcc produced by CS snails (Table 2). In the case of *P. daubneyi,* brown or blackish mcc observed just after cercarial encystment might be cercariae which died during or just after this process and rapidly became dark in the following hours. Their presence in mcc production seems less prejudicial for mcc collection because they can be differentiated from other mcc and only represent 2.6% (population A) or 2.9% (population B) of mcc coming from CS snails in temperature-challenged groups (Table 3).

In conclusion, the use of regular temperature changes for infected *G. truncatula* during the patent period gives a maximum number of snails which shed their cercariae in the case of *F. hepatica* or *P. daubneyi*; and enhanced mcc production by CS snails. However, this method also results in production of incompletely formed mcc which are sometimes difficult to identify, especially for *F. hepatica*, in reason of their probably limited viability. Mcc collection from temperature-challenged snails for infecting ruminants must be carefully carried out to avoid eventual problems of non-infection in these definitive hosts. Moreover, as daily temperature fluctuations in nature also had a positive effect of mcc production [9], it seems useful to determine the proportion of incompletely formed mcc produced by naturally-infected *G. truncatula* because all populations of this species did not have the same susceptibility towards local *F. hepatica* or *P. daubneyi* [17].

## References

[1] Abrous M, Rondelaud D, Dreyfuss G. 1999a Influence of low temperatures on the cercarial shedding of *Paramphistomum daubneyi* from the snail *Lymnaea truncatula*. Parasite, 6, 85–88 10229944 10.1051/parasite/1999061085

[2] Abrous M, Rondelaud D, Dreyfuss G. 1999b *Paramphistomum daubneyi* and *Fasciola hepatica*: influence of low temperatures on the shedding of cercariae from dually infected *Lymnaea truncatula*. Parasitology Research, 85, 765–769 10431746 10.1007/s004360050628

[3] Andrews SJ. 1999 The life cycle of *Fasciola hepatica*, in Fasciolosis, Dalton JP, Editor.CABI Publishing: Oxon, 1–29

[4] Audousset JC, Rondelaud D, Dreyfuss G, Vareille-Morel C. 1989 Les émissions cercariennes de *Fasciola hepatica* L. chez le mollusque *Lymnaea truncatula* Müller. A propos de quelques observations chronobiologiques. Bulletin de la Société Française de Parasitologie, 7, 217–224

[5] Caron Y, Lasri S, Losson B. 2007 *Fasciola hepatica*: a study of the vectorial capacity of four different species of lymnaeid snails commonly found in Belgium. Veterinary Parasitology, 149, 95–103 17697752 10.1016/j.vetpar.2007.07.012

[6] Dixon KE. 1966 A morphological and histochemical study of the cystogenic cells of the cercaria of *Fasciola hepatica* L. Parasitology, 56, 287–297 4225454 10.1017/s0031182000070876

[7] Graczyk TK, Fried B. 1999 Development of *Fasciola hepatica* in the intermediate host, in Fasciolosis. Dalton JP, Editor.CABI Publishing: Oxon, p. 31–46

[8] Hodasi JKM. 1972 The output of cercariae of *Fasciola hepatica* by *Lymnaea truncatula* and the distribution of metacercariae on grass. Parasitology, 65, 431–436 10.1017/s0031182000044644 5010460

[9] Kendall SB, McCullough FS. 1951 The emergence of the cercariae of *Fasciola hepatica* from the snail *Lymnaea truncatula*. Journal of Helminthology, 25, 77–92

[10] Mercer EH, Dixon KE. 1967 The fine structure of the cystogenic cells of the cercaria of *Fasciola hepatica* L. Zeitschrift für Zellforschung und Mikroskopische Anatomie, 77, 331–344 4233864 10.1007/BF00339239

[11] Mud Snail Study Group *Omphiscola glabra* captive breeding programme*.* Release strategy, 2006, Website: http://www.ephemeroptera.pwp.blueyonder.co.uk/mssg/1869 (Consulted on March 22, 2012)

[12] Ollerenshaw CB. 1971 Some observations on the epidemiology of fascioliasis in relation to the timing of molluscicide applications in the control of the disease. Veterinary Record, 88, 152–164 5102169 10.1136/vr.88.6.152

[13] Rondelaud D, Vignoles P, Dreyfuss G. 2009 La Limnée tronquée, un mollusque d’intérêt médical et vétérinaire. PULIM: Limoges

[14] Rondelaud D, Fousi M, Vignoles P, Moncef M, Dreyfuss G. 2007 Optimization of metacercarial production for three digenean species by the use of Petri dishes for raising lettuce-fed *Galba truncatula*. Parasitology Research, 100, 861–865 17061111 10.1007/s00436-006-0353-2

[15] Smyth JD, Halton DW. 1983 The physiology of trematodes. Cambridge University Press: Cambridge

[16] Taylor EL. 1965 Fascioliasis and the liver-fluke, 64 FAO Agricultural Studies: Roma

[17] Vignoles P, Dreyfuss G, Rondelaud D. 2002 Larval development of *Fasciola hepatica* in experimental infections: variations with populations of *Lymnaea truncatula*. Journal of Helminthology, 76, 179–183 12015832 10.1079/JOH2002112

[18] Vignoles P, Alarion N, Bellet V, Dreyfuss G, Rondelaud D. 2006 A 6–8 day periodicity in cercarial shedding occurred in some *Galba truncatula* experimentally infected with *Fasciola hepatica*. Parasitology Research, 98, 385–388 16374617 10.1007/s00436-005-0078-7

